# How much is policy driving the adoption of cover crops? Evidence from four EU regions

**DOI:** 10.1016/j.landusepol.2022.106016

**Published:** 2022-05

**Authors:** Jonas Kathage, Bert Smit, Bas Janssens, Wiepie Haagsma, Jose Luis Adrados

**Affiliations:** a European Commission, Joint Research Centre, Spain; b Wageningen Economic Research, the Netherlands; c Wageningen Plant Research, the Netherlands; d Kantar TNS, Spain

**Keywords:** Agricultural policy, Climate change, Cover crops, Environment, Farm survey, Greening, Nitrates Directive, Technology adoption, Willingness to accept

## Abstract

EU agriculture is facing increasing expectations and pressure from society and policymakers to support environmental protection and climate change mitigation. Catch and cover crops (CCC) are an underused farming practice that can potentially contribute towards these goals. Previous research is sparse and has yielded few relevant insights into CCC adoption behaviour by farmers. In this study we analyse a dataset from farm surveys in four EU regions to better understand the role of policy and non-policy factors in CCC adoption. Our data suggests that adoption rates vary widely between regions, while farm adoption intensities are low. We find that policy is by far the strongest determinant of adoption rates and adoption intensities. CCC adoption patterns have been shaped mainly by the Nitrates Directive and the Common Agricultural Policy's greening requirements. Agronomic motives are a third but much weaker impetus for adoption. Environmental and climate change considerations do not play a significant role in farmers' adoption decisions. Most non-adopters would likely become adopters if stronger policy obligations or additional subsidies were implemented. Non-adopters‘ responsiveness to subsidies and willingness to accept is highly varied but only weakly predictable from easily observed farm characteristics.

## Highlights

• Catch and cover crops (CCC) may serve environmental protection and climate change mitigation goals.
• We analyse a dataset from farm surveys in four EU regions to understand the role of policy and other factors in CCC adoption.
• We find that policy is by far the strongest determinant of adoption rates and adoption intensities.
• Environmental and climate change considerations do not play a significant role in farmers' adoption decisions.
• Most non-adopters would likely become adopters if stronger policy obligations or additional subsidies were implemented.

## 1 Introduction

The European Commission has identified climate change mitigation in agriculture as one of the main priorities for the Common Agricultural Policy (CAP) after 2020. The Commission has also proposed a new delivery model which emphasises performance, and foresees more subsidiarity. Successfully aligning the CAP with climate action requires a good understanding of the mitigation potential of available agricultural practices and technologies, as well as of the policy measures that can potentially lead to a higher use of promising but underutilised practices and technologies.

Catch and cover crops (CCC) are among the practices under discussion that may contribute to enhanced environmental and climate performance of the CAP, and have already been part of the 2014–2020 CAP. CCC have several potential beneficial effects on agronomic and environmental outcomes (Blanco-Canqui et al., 2015, Valkama et al., 2015, Alvarez et al., 2017, Marcillo and Miguez, 2017, Daryanto et al., 2018, Osipitan et al., 2018, Thapa et al., 2018). CCC may also help mitigate climate change by increasing carbon sequestration and albedo, and by reducing greenhouse gas emissions associated with fertiliser use (Poeplau and Don, 2015, Kaye and Quemada, 2017, Carrer et al., 2018). However, adoption of CCC by farmers is limited in many countries and regions where their wider cultivation is at least technically possible, including North America and Europe (Eurostat, 2010, Bergtold et al., 2019).

Limited benefits (Seifert et al., 2018) and potential negative effects including economic cost (Bergtold et al., 2019) and other disadvantages (e.g. management challenges) may account for lack of adoption. However, only a limited amount of empirical research aimed at explaining the often low adoption of conservation practices is available, and mostly concerns the US and not Europe (Wauters and Mathijs, 2014, Arbuckle and Roesch-McNally, 2015). A small but increasing number of studies is available regarding CCC, again most of them for the US (e.g. Myers and Watts, 2015; Dunn et al., 2016; Lira and Tyner, 2018; Roesch-McNally et al., 2018) and few on Europe (e.g. Werner et al., 2017).

In order to increase technology adoption and design efficient policy interventions, it is necessary to identify potential reasons for non-adoption and rank them by their relative importance (Wreford et al., 2017). In general, two different interpretations of low technology adoption can be distilled from the literature: First, adoption would be beneficial but is not happening because farmers are not aware of the practice or how to implement it, or are prevented from adoption by internal (e.g. irrational behaviour) or external (e.g. lack of access to credit) constraints. In this interpretation, adoption is often assumed to be beneficial. The second interpretation is that adoption would not be beneficial to farmers and non-adoption is not fundamentally the result of constraints (Kathage et al., 2015, Seifert et al., 2018, Bergtold et al., 2019).^2^ Note that in both interpretations, policy interventions can increase adoption: In the first case, policy has to remove the constraints (but not change the inherently beneficial nature of adoption). In the second case, policy has to raise the benefits from adoption, typically through subsidies or cost-sharing (Conner et al., 2016).

One complication to these interpretations is that the use of technologies and practices may have externalities. For example, carbon sequestration is a positive externality of CCC cultivation because most of the benefits from climate change mitigation are not captured by the farmer. Therefore, non-adoption may be inefficient from a societal perspective even if the farmer is better off without adopting. The literature is characterised by a frequent lack of clearly distingushing between the overall benefits of adoption and the benefits of adoption to farmers (e.g. Blanco-Canqui et al., 2015; Carlisle, 2016), with few exceptions (e.g. Snapp et al., 2005). On the other hand, beliefs about the external environmental effects of a technology may form part of the farmer's utility function and be among the reasons for or against adoption (Stupak et al., 2019, Wuepper, 2019). This can happen through internal motives (e.g. environmentalist mindset) and social norms and pressures (Wauters and Mathijs, 2013, Carlisle, 2016, Dessart et al., 2019).

The understanding of why non-adopters do not use CCC can be enhanced with knowledge about the reasons why adopters use CCC. Importantly, these reasons may include existing policies, and may inform future policy design. Therefore, it is crucial to analyse adoption and non-adoption together.

Classical adoption studies typically apply some form of regression analysis on datasets composed of adopters and non-adopters to estimate the determinants of adoption (e.g. Bergtold et al., 2012; Sánchez et al., 2016). But at least in the case of conservation practices, these studies have not produced consistent results, which makes it difficult to draw clear lessons for policy (Wauters and Mathijs, 2014). One of the reasons for conflicting findings is omitted variable bias. Another reason is low external validity. A more severe problem of many adoption studies is the issue of internal validity, since regressions without proper instrumentation only show correlations, not causality. Non-regression methods that are also correlational in nature (e.g. Bijttebier et al., 2018) have the same problem: Adopters may differ from non-adopters in a number of characteristics and beliefs, but that does not mean that any of these variables are causally related to adoption.

Based on data from farm surveys in four EU NUTS 2 regions in France, the Netherlands, Romania and Spain, this study adds to the small number of research publications on the adoption of CCC in Europe. We also avoid the other above-mentioned weaknesses of previous work: We directly ask adopters in an open question about the main reason for adoption. This tackles the issues of causality and omitted variable bias. It also allows us to evaluate the effect of policies versus other proposed determinants, including environmental motives. We compare the data from this open question with other relevant information from the survey, which strenghtens our findings. We complement our examination of the reasons for and against adoption with an analysis of how responsive non-adopters are to hypothetical additional subsidies for CCC cultivation. In particular, we investigate whether any farm or farmer characteristics can predict (serve as proxies for) non-adopters’ responsiveness to subsidies and its extent (willingness to accept, WTA). In the next section, we describe the empirical approach we used for the survey and data analysis. We present the results by country in Section 3. Section 4 discusses the main findings of the study, and Section 5 presents the conclusions, including lessons for policy.

In the EU, special attention must be paid to policy as a factor in adoption. Before going into the next section, we briefly summarise two EU policies that are among the potential drivers of CCC cultivation: the Nitrates Directive and the CAP. The Nitrates Directive (1991) aims to protect water quality across Europe by preventing nitrates from agricultural sources polluting ground and surface water and by promoting the use of certain farming practices. The Directive is implemented by Member States (MS), who establish Action Programmes that are compulsory in designated Nitrate Vulnerable Zones (NVZ). The cultivation of CCC has been included in Action Programmes by several MS at different points in time and with different provisions, including France and the Netherlands.

Since 2015, the greening obligations of the Common Agricultural Policy require farmers with more than 15 ha to maintain at least one type of Ecological Focus Area (EFA) on a certain share^3^ of their arable land (or face penalties). National governments have to draw up lists of eligible EFA options (from a common EU list), from which their farmers can choose one or several options to fulfill the obligation with. The common list of EFA options includes catch crops/green cover (chosen by France, the Netherlands and Romania) and also nitrogen-fixing crops (chosen by all four case study countries). Farmers are exempt from the EFA requirement in a number of circumstances; notably if more than 75% of their agricultural area is grassland. Several MS and regions (including France, Romania and Spain) have also included some forms of CCC in Rural Development Programmes (RDP) of the CAP. In contrast to the obligatory NVZ practices and greening, cultivation of CCC under RDP is voluntary and offers farmers additional subsidies.

## 2 Empirical approach

The four case studies were selected on the basis of the current adoption and mitigation as well as the future adoption and mitigation potential they offer (see Smit et al., 2019 for more details on the research project). We defined adoption potential as the size of the area where CCC are not yet adopted but adoption is feasible. Mitigation potential was defined as the amount of greenhouse gases that are sequestered and avoided by using CCC compared to not using CCC. For each NUTS 2 region, the mitigation potential and the adoption potential were estimated for each crop group. The mitigation potential (in kg CO_2_e/ha) for each region r was defined as follows:

Mitigation potential_r_ = Mitigation potential from N_r_ + Mitigation potential from C_r_,

where N refers to the mitigation potential resulting from the amount of N-fertiliser avoided, and C refers to the mitigation potential resulting from carbon sequestration. The mitigation potential was estimated only based on carbon sequestration, as this is the major of source of mitigation.^4^ Disregarding the contribution of reducing N-losses and N-fertiliser input results in an underestimation of the mitigation potential, but has little influence on the ranking of case study regions with a high potential.

The adoption potential was calculated as the total number of hectares of three big crop groups (cereals, industrial crops and green maize) per NUTS 2 region, but corrected for the area on which CCC were already grown in 2010, according the latest Eurostat data available at the time the research was conducted (Eurostat, 2010). In regions with a high share of cereals and a low current adoption rate of CCC, the potential to adopt (more) CCC is high. Besides these three big crops, permanent crops (mainly olives and vineyards) were also taken into consideration (olives were later dropped because of the limited feasibility of growing CCC). Other crops can be important in certain regions or MS, but their acreage was small when compared to the three big crops and the two permanent crops listed. Since acreage of a crop is dominant in the calculation of the adoption potential, other crop groups than these five were disregarded in the selection of case study regions.

We decided to focus on four case study regions, each with 150 farmers interviewed. The ranking of NUTS 2 regions by the estimated mitigation and adoption potentials formed the basis for the case study selection. In addition, the selection process aimed at including a diversity of climatological and legislative conditions and farm types, and also considered the varying regional costs of conducting surveys. Three case studies were selected with a high mitigation potential and a low (expected) adoption rate (Centre, France; Sud-Muntenia, Romania; Castile and Leon, Spain). One case study with a high mitigation potential and a high (expected) adoption rate was selected (Overijssel, the Netherlands).

The original target for each survey was to include an equal number of adopters and non-adopters. However, a pre-test revealed that it was very difficult to find non-adopters in the French and Dutch regions. Therefore, the restriction of a 50% maximum share of adopters in the sample was dropped for these regions.

A questionnaire was developed and included three blocks: First, for those farmers adopting CCC, questions related to the CCC practices implemented in detail (species, rotation, planting dates, agronomic practices, use of CCC after termination). Second, for all farmers, questions related to reasons for or against the adoption of CCC. Questions could be different for adopters and non-adopters, but the issues covered were similar. The third block, for all farmers, was composed of questions about general farm and farmer characteristics (e.g. age, education, economic size, types of operations, etc.). A preliminary questionnaire was first tested with a few farmers in all regions. The results from this test were used to establish the final version of the questionnaire.

Interviewers were trained to better understand the objectives of the survey and the topic of CCC. Arable farmers in the selected regions were invited to participate in the survey. The main contact points were cooperatives and farm input suppliers, and also networks of advisors and extension services. The target population included those who grew what we refer to as target crops: cereals (wheat, barley, grain maize, triticale, rye, oats, spelt), oilseed rape, sunflower, soybeans or green maize/silage maize. Small farms with a farm size of up to 3–6 ha (country-dependent, based on a Standard Output of €1250) were excluded. The surveys were completed in the summer of 2018.

Adoption rates were estimated through the following procedure: the interviewer first asked whether the farmer knew what CCC are. Following the farmer's answer, the interviewer provided the following definition of CCC:

*Cover and catch crops are grown primarily to fulfil certain functions such as to reduce leaching, to provide nitrogen to the next crop, to reduce soil erosion, to improve soil structure, soil fertility and soil water properties, to reduce pest pressure on crops, to prevent weed growth, and/or to increase the biodiversity of the farming landscape and environment. CCC are living plants intentionally sown by the farmer. Finished CCC are usually not sold, but terminated and left on the field or ploughed in, although they might also be harvested and used as animal feed or for bioenergy production. CCC are often grown in between main / cash and feed crops, but can also be undersown. Many species can be grown as CCC, for example annual or perennial grasses, brassicas and mustards, legumes or others like linen and buckwheat; and often, mixes of different species are grown*.

After making sure that the farmer understood the concept of CCC, the next question was if he/she was an adopter or non-adopter. The interviewer recorded the answer and checked whether the maximum quota for adopters had already been reached. If this was the case and the farmer was an adopter, the interviewer would thank the farmer for his or her willingness to participate and explain that the quota for adopters had already been reached. He would then ask him/her if he/she knew a person who is a non-adopter. If the quota had not yet been reached, the interviewer would complete the remainder of the questionnaire. An estimation of the adoption rate in the target population was made based on the numbers of adopters and non-adopters contacted (the selection of which was random, hence representative of the target population), not the numbers of adopters and non-adopters with whom full interviews were conducted (influenced by the quotas and hence not representative).

We answer our main research question about the determinants of adoption and non-adoption by analysing and comparing the data coming from relevant survey questions, and using logical reasoning to reach conclusions. We use standard mean comparisons and correlational analysis to examine whether non-adopters’ responsiveness to hypothetical subsidies is predicted by any farm or farmer characteristics. In the next section, we present and discuss the results, first on the reasons for and against adoption (by country), and second on the predictors of the responsiveness to subsidies.

## 3 Results

### 3.1 Reasons for and against adoption

#### 3.1.1 Centre (France)

In the French^5^ case study, 397 farmers were contacted. Of them, 332 were adopters and 65 non-adopters. To achieve the most balanced sample possible, it was attempted to interview all non-adopters, but due to some refusals, 42 non-adopters were fully interviewed, plus 120 adopters, reaching a total sample of 162 farmers. Based on the numbers of contacted adopters and non-adopters, the adoption rate in the case study region was estimated to be 83.6% (332 out of 397). The average farm adoption intensity (CCC hectares over total agricultural hectares) among French adopters was 18%. The target crop adoption intensity (CCC hectares over target crop hectares) was 25%.

The most frequently mentioned reasons for adoption among the French adopters are related to policy (60%), followed by agronomy (33%). Environment, harvest, pest management and other reasons are less frequent (Table 1). Interestingly, obligation is the exclusive defining aspect of policy for all 60% of farmers (Table A1). Subsidies are never mentioned. Regarding the 33% of farmers with agronomy as main reason, soil improvement is most frequently mentioned (27%).

**Table 1:** Main reason for CCC adoption, grouped (% of adopters, by region).

	Centre (France)	Overijssel (Netherlands)	Sud-Muntenia (Romania)	Castile and Leon (Spain)
Policy	60.0	54.4	83.3	15.4
Agronomy	32.5	49.7	21.8	65.4
Environment	11.7	12.8	–	7.7
Harvest	10.8	4.7	–	7.7
Pest management	3.3	2.0	–	7.7
Other	9.2	1.3	2.6	3.8
n	120	149	78	26

Note: Main reason as spontaneously stated by farmer (open question, answers coded into dummies, disaggregated results in Table A1). Columns may add to more than 100% because farmers sometimes mentioned several reasons.

Farmers' responses to a question on the inclusion of CCC in (obligatory and/or voluntary) policies are consistent with the view that policy is a main driver of adoption (Table 2). Sixty-nine percent of adopters perceive that CCC adoption is linked to mandatory policies, 32% to voluntary policies. Only 8% of adopters see no connection of CCC with mandatory or voluntary policies. When asked to identify the policies they had in mind, 38% of farmers mentioned (rules associated with) the CAP (e.g. greening or EFA) and 17% (rules associated with) the Nitrates Directive (Table 3). Many farmers (43%) made unclear statements about the policy they were referring to, indicating that despite a general perception of policies promoting CCC, detailed knowledge of these policies is limited. Interestingly, not only is CCC cultivation seen as obligatory by most of the farmers who mention the Nitrates Directive: Also, 70–80% of farmers who mention the CAP consider CCC as being obligatory under it.

**Table 2:** CCC cultivation under schemes/policies (% of adopters).

	Centre (France)	Overijssel (Netherlands)	Sud-Muntenia (Romania)	Castile and Leon (Spain)
Mandatory	69.2	85.9	87.2	34.6
Voluntary	31.7	8.1	11.5	61.5
Neither mandatory nor voluntary	8.3	6.0	5.1	3.9
n	120	149	78	26

Note: Multiple answers possible

**Table 3:** Identification of policies promoting CCC cultivation (% of adopters).

	Centre (France)	Overijssel (Netherlands)	Sud-Muntenia (Romania)	Castile and Leon (Spain)
CAP	37.5	28.5	82.1	50.0
Nitrates Directive	17.4	61.7	–	–
Organic rules	1.7	0.7	–	–
Other policies	4.2	–	–	–
Unclear answer	42.5	0.8	12.8	46.2
n	120	149	78	26

Note: Columns may add to more than 100% because multiple answers were possible. Columns may add to less than 100% because of missing answers.

Further questions about the greening (EFA) obligation support the hypothesis that the CAP is driving CCC cultivation in the French case study. Eighty percent of adopters grow CCC because of the greening subsidies, although 38% said that other reasons besides greening are also relevant (Table 4). Some of these other reasons could be related to the Nitrates Directive, but so do some of the non-policy (mostly agronomic) reasons.

**Table 4:** Greening as a reason for CCC adoption (% of adopters).

	Centre (France)	Overijssel (Netherlands)	Sud-Muntenia (Romania)	Castile and Leon (Spain)
Yes, to get EFA subsidies (only)	42.5	13.4	85.9	50.0
Yes, to get EFA subsidies (+other reasons)	37.5	52.4	12.8	30.8
No, not for EFA. Use other EFA measures	6.7	4.0	–	3.9
No, not for EFA. No other EFA measures	1.7	–	–	3.9
No, EFA not obligation for my farm	8.3	21.5	1.3	11.5
No, grow CCC for other subsidies/obligations	3.3	8.7	–	–
n	120	149	78	26

It is important to recognise that even if CCC are mandatory or voluntary under a certain policy, this policy is not necessarily the reason for adoption. Farmers could have non-policy reasons for adoption, in the case of which the policy has no impact on adoption ('deadweight'). As a further test of the relevance of greening for adoption, we asked adopters if they would grow less or no CCC in a hypothetical scenario in which CCC was not included among the EFA options (Table 5). More than half (52%) would grow less or no CCC. Almost half of adopters say they would maintain the same level of CCC cultivation without greening, which can be explained by the Nitrates Directive and non-policy reasons.

**Table 5:** CCC area change without EFA option (% of adopters).

	Centre (France)	Overijssel (Netherlands)	Sud-Muntenia (Romania)	Castile and Leon (Spain)
Same	48.3	73.8	24.4	73.1
Less	33.3	16.8	34.6	7.7
None	18.3	9.4	41.0	19.2
n	120	149	78	26

**Fig. 1:**
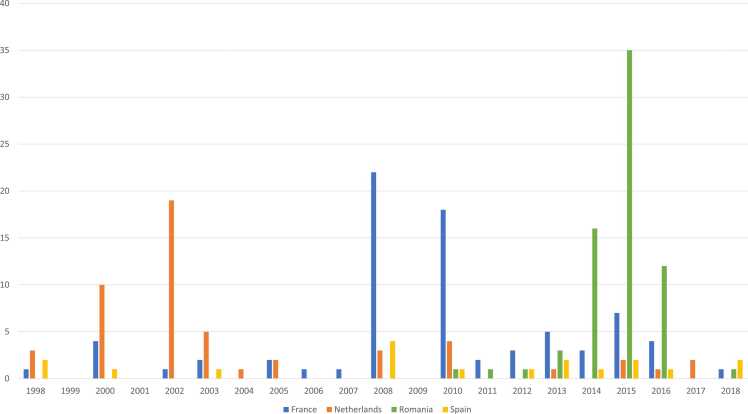
CCC adoption starting year (number of adopters).

The conclusion that both greening and Nitrates Directive matter as drivers for CCC adoption is supported by an examination of the years when CCC were first adopted (Fig. 1). Most adopters started around the year 2009, which is when a catch crop obligation in the autumn before spring crops was introduced as part of the fourth Nitrates Directive Action Programme in France. The second, but smaller peak is centered around the year 2015, when the greening obligation was first introduced.

While our data suggests a high CCC adoption rate of 84% and identifies greening as an important driver of it, greening can also help explain the low CCC adoption intensities we found. CCC adopters grow CCC on an average of only 18% of their agricultural area, which is close to the approximately 17% minimum EFA requirement for catch crops/green cover. Only 20% of farmers have farm adoption intensities above 25%. However, the Nitrates Directive may also be a reason for limited adoption intensities, as it mandates a catch crop only in the autumn before spring crops (not before winter crops, which are more common). Apart from these policy-related reasons for limited adoption intensity, non-policy reasons may also matter. Fifty-eight percent of adopters affirmed that there were problems or risks limiting them from growing CCC on a larger area. Farmers frequently stated weather risks (lack of water), cost of CCC cultivation and time requirements as limiting factors.

We now turn to non-adopters and their reasons for not growing CCC. Asked to assign importance to potential reasons from a pre-defined list, non-adopters frequently identified as the most important reason a lack of benefits from CCC, high costs of cultivation, and unsuitability of CCC for their crop rotation. Similarly, lack of profitability, lack of water, and unsuitability for the crop rotation were stated by former adopters (who constitute 43% of non-adopters) as the main reasons they stopped growing CCC.

Could policy be modified to change the incentives of non-adopters in favour of adopting CCC? The uninteresting case of an obligation with high non-compliance penalties aside, how much in additional subsidies would be required for turning non-adopters into adopters? Seventy-four percent of non-adopters affirmed that they would grow CCC if they were given subsidies for it. This result is in line with high cost of cultivation and lack of profitability as main reasons for non-adoption. Only a minority of 26% of non-adopters said subsidies would not change their mind, which could be related to water, labour, or crop rotation constraints. The 74% of non-adopters with a willingness to adopt (given subsidies) indicated a range of necessary subsidies (WTA), with a mean of €176/ha (25th percentile: €60, p75 = €250). To put this number into perspective, it is somewhat lower than the average CAP direct payments in France (around €260/ha, EC, 2018). The mean WTA is signficantly higher than the mean cost of cultivation (€94/ha) reported by adopters (Smit et al., 2019, p. 29). The source of the discrepancy between WTA and cost of cultivation is not clear, but one should note that the French WTA is similar to the WTA in other countries, while the French cost of cultivation is significantly lower than the cost of cultivation in other countries.

#### 3.1.2 Overijssel (The Netherlands)

In the Dutch case study area, which was selected for its high expected adoption rate, it was difficult to find non-adopters. Therefore, interviews in the originally selected province of Overijssel were supplemented with interviews in the neighbouring province of Flevoland. Even with this supplementation, only two non-adopters were encountered and interviewed, out of a total of 151 interviewed farmers (149 adopters). Hence, the estimated adoption rate in the case study area is 98.7%.

The average farm adoption intensity was 20%, similar to the French case. In contrast, the average target crop adoption intensity was 92%. The reason for this much higher target crop adoption intensity is that Dutch farmers grow large parts of their land with grass, which is not among the target crops defined in our study. The main target crop cultivated by the Dutch farmers is maize, grown on a small share of the farmland. The high share of (mostly permanent) grassland is linked with a derogation under the Nitrates Directive. This derogation allows farmers with large shares of grassland to apply a larger amount of nitrogen from manure per hectare. Many CCC adopters have dairy cows and make use of the derogation because of the otherwise high cost of manure disposal.

The most frequently mentioned groups of reasons for adoption among the Dutch adopters are policy (54%) and agronomy (50%). Environment, harvest, pest management and other reasons are much less frequent, as in the French case (Table 1). Obligation is practically the only mentioned reason in the policy group of reasons (Table A1). Subsidies are only mentioned by 1% of adopters. Among the 50% of farmers with agronomy as the main reason, soil improvement is the most frequently mentioned reason (46%).

Interpreting CCC cultivation as an obligation accords with the fact that 86% of Dutch adopters associate CCC adoption with a mandatory policy (Table 2). Only 8% see adoption under the relevant policy as voluntary, a much lower figure than in France. When asked to identify the policies, 62% referrred to rules associated with the Nitrates Directive (Table 3). Twenty-nine percent of adopters mentioned elements belonging to the CAP (such as greening). Unclear answers were extremely rare, suggesting a high level of knowledge of the relevant policies. CCC cultivation is seen as obligatory by most farmers who mention the Nitrates Directive. Also, over 80% of farmers who mention the CAP see CCC as obligatory under it. These last two results are similar to the French case study.

The relatively large share (50%) of adopters citing agronomic reasons for adoption may appear to be in conflict with the hypothesis that most CCC adoption is driven by policy obligations. CCC adoption may indeed be obligatory, but an obligation is not a necessary condition for adoption if farmers would also grow CCC without the obligation. Conversely, the agronomic benefits of a mandatory and widespread practice may simply be the first thing on farmers' minds, when the real driver is a policy obligation. We return to this issue after considering the remaining relevant evidence.

Data from further questions confirm the weaker role of the CAP in driving CCC cultivation. While 66% of adopters grow CCC because of the greening subsidies, only 13% said that greening was the only relevant reason (Table 4). Other reasons besides greening (likely the Nitrates Directive and agronomic reasons) were thus important for more than half of the adopters. For a significant remainder (34%) of adopters, the reasons for adoption are unrelated to greening. Most adopters (74%) would grow the same amount of CCC if CCC was not among the EFA options (Table 5).

In line with this, almost all Dutch adopters first started growing CCC many years before greening was introduced (Fig. 1). The peak starting year is 2002. This time period coincides with the introduction of a catch crop obligation after maize on certain soil types in the Dutch Nitrates Directive Action Programme.

The observed CCC farm adoption intensities of around 20% can also be explained by policy: Many adopters grow at least 80% of their area with grass (without which they would not be allowed to make use of the derogation under the Nitrates Directive). The remaining 20% are typically sown with maize, after which a catch crop is obligatory under the Nitrates Directive. Greening is less important: Smaller farms with a high share of grassland are exempted from the EFA obligation. Larger farms with the EFA obligation can fulfill it with CCC on about 17% of their area, but in case they already grow that amount of CCC due to the Nitrates Directive obligation, the EFA obligation has equally no effect on CCC adoption or alternative EFA options (deadweight).

Most adopters have farm adoption intensities of 20% or less, and only 10% of adopters have adoption intensities above 23%. This suggests that given the policy regime, there is little incentive for farmers to grow more CCC. Indeed, 48% of adopters say there are problems or risks preventing them from expanding CCC cultivation; weather conditions (e.g. too wet after main harvest) are mentioned most frequently. The 52% of adopters who are not facing problems or risks limiting CCC expansion are probably not much interested in growing more CCC in the first place. Furthermore, many farmers grow grass not in rotation but as permanent grassland. Permanent grassland does not have rotation 'gaps' and therefore allows no CCC cultivation. Adoption intensities would probably increase if farmers were allowed to make use of the derogation with less grassland, because with less grassland, maize would most likely expand (and with it the CCC obligation).

We now return to the question of agronomy versus policy as adoption drivers for those adopters who indicated agronomy as the main reason for growing CCC.

Unfortunately, we have no direct information on whether these farmers have a CCC obligation under the Nitrates Directive. Indirectly, we can make an educated guess: About 80% of these adopters grow a main crop on 20 ha or less. Over 90% grow arable crops and also have grassland, and all of them have livestock. This makes it likely that these farmers grow maize on at least some part of the remaining farm area (for feed). Since they are located in the same region, most of them are likely to grow maize on sandy soils, which would imply a CCC obligation (under the Nitrates Directive).

Answers from this group of adopters to the question on the relevance of the EFA requirement for CCC adoption shows that almost two-thirds of them grow CCC to fulfill the EFA requirement, but also for other reasons (the remainder mostly do not face the EFA requirement or indicate they grow CCC for other reasons or obligations). Also, 90% would continue to grow the same amount of CCC without the EFA requirement. In summary, the question over the primacy of either agronomy or policy cannot be answered with full certainty. However, given the data discussed in the previous paragraph and the rest of this section, it is unlikely that most of the farmers in this group would grow CCC without the Nitrates Directive.

The number of non-adopters in the sample is only two, too small to draw reliable inferences.

#### 3.1.3 Sud-Muntenia (Romania)

In Sud-Muntenia, a total of 243 farmers were contacted. Of them, 112 were adopters and 131 were non-adopters, indicating an adoption rate in the region of 46.1%. Full interviews were conducted with 78 adopters and 77 non-adopters. The average farm adoption intensity was 14%, not far from what we observed in France and the Netherlands. The average target crop adoption intensity was only slightly higher (16%), indicating that most of the farm area is grown with target crops.

The most frequently mentioned reason for adoption is policy (83% of adopters), far ahead of agronomy (22% of adopters). Other reasons do not play a role. Policy thus appears even more powerful in explaining adoption in Romania than in France and the Netherlands. An obligation is defining the policy reason for 67% of adopters (Table A1). Interestingly, 22% cite subsidies, a significantly larger share than in France and the Netherlands.

Eighty-seven percent of adopters consider CCC as being mandatory under a policy, a similar figure as in the Netherlands (Table 2). Twelve percent regard CCC cultivation as being voluntary under a policy. When asked to identify the policies, most farmers named the Agency for Payments and Intervention in Agriculture (APIA), which is reponsible for implementation and controls of the CAP in Romania (Table 3). Therefore, the relevant policy is likely to be the CAP.

To the question about the relevance of greening for CCC adoption, 86% of adopters answered they grow CCC only because of the EFA requirement (Table 4). Thirteen percent grow CCC because of the EFA requirement, but also for other reasons. The overwhelming importance of greening is confirmed by data showing that 76% of farmers would grow less or no CCC if CCC were not an option for fulfilling the EFA requirement, including over 40% who would stop growing CCC entirely (Table 5). Almost all adopters first started growing CCC around the year 2015, when greening was introduced (Fig. 1).

Greening can also help explain the low CCC adoption intensities we found. CCC adopters grow CCC on an average of only 14% of their agricultural area, and 83% of adopters grow CCC on 20% or less of their area. This is close to the EFA requirement of 17% for the catch crops/green cover option. Futhermore, most adopters (81%) indicated that there were no problems or risks limiting them from growing CCC on a larger area.

We now analyse non-adoption. Non-adopters frequently identified as the most important reason high costs of cultivation, no benefits, lack of availability of water, labour and machinery, unsuitability of CCC for their crop rotation, and lack of awareness of CCC (53% of non-adopters were not familiar with CCC). It is quite possible that the CCC adoption rate would increase if some of these constraints were absent. After all, many non-adopters face the EFA requirement and could potentially fulfill it with CCC. On the other hand, the data from adopters show little evidence for non-policy drivers of adoption, and non-adopters may continue to prefer to fulfill the EFA obligation with other options.

Further data reveal that the main constraint is monetary and can potentially be overcome by policy: Over 80% of non-adopters say they would start growing CCC if they were given subsidies for it. While there is a range of subsidies that would be required (WTA), the mean is €154/ha (p25 = €126, p75 = €210). These amounts are similar to the average direct payments in Romania (about €200/ha, EC, 2018), and similar to the average cost of cultivation reported by Romanian adopters (€153/ha, see Smit et al., 2019, p. 29). There is no statistically significant difference from France in mean WTA.

#### 3.1.4 Castile and Leon (Spain)

In the Spanish case study region, 174 farmers were contacted and 155 fully interviewed. During the course of the field work, no CCC cultivation was found among farms without irrigation (probably because of insufficient rainfall). Since the intention was to find a sizeable number of adopters, farms with irrigation were then purposefully oversampled. Ninety farms in the final sample had irrigation, and the CCC adoption rate among those irrigated farmers was 28.9% (26 adopters). In order to estimate the adoption rate among the overall target population (i.e. including non-irrigated farms), a correction was made: Irrigated farms account for 40% of all farms with more than 5 ha in the region. Therefore, the corrected adoption rate estimate is 28.9% of 40%, i.e. 11.6%. The average farm adoption intensity was 18%, similar to the other case studies. The target crop adoption intensity was higher with 26%, similar to the French case study.

Agronomy was the most important reason for adoption (65% of adopters). Policy was the second most frequently cited reason, but only relevant for 15%. The environment, harvesting and pest management are less important individually, but together add up to 23% of adopters. Unlike in the other case studies, soil improvement accounts for less than half of the total in the agronomy group of reasons (Table A1). N fixation and improving the next crop are together more frequently cited. Obligation is the most mentioned reason in the policy group of reasons.

The low importance of obligatory policies is confirmed by the fact that the majority (62%) of adopters regarded CCC cultivation as voluntary under policies (Table 2). This is a notable difference from the three other case studies, where CCC cultivation was mostly seen as obligatory under policy. Half of adopters cited the CAP (or elements of it like greening), the other half gave unclear answers, including no policy (Table 3).

Curiously, most (81%) adopters said they grow CCC because of the EFA requirement, with 50% because of it only and for no other reason (Table 4). As in the Dutch case study, an apparent conflict between agronomy and policy as the primary driver of adoption emerges. All Spanish adopters grow legumes as CCC, which are one of the EFA options. However, responses to a further question about the importance of greening for adoption suggest that most (73%) adopters would grow the same amount of CCC without the EFA requirement (Table 5). The contradiction between this question's answer pattern and the responses to the previous question can only be resolved by allowing one of the two questions to be at least partly discounted. We believe that the question on EFA requirement as a reason for adoption should be discounted, for three reasons 1) it is more complex and hence more likely to be misunderstood, 2) most adopters indicated agronomy as the main reason for adoption and policy only rarely, 3) the years adopters first started growing CCC are dispersed and not centered on the introduction of greening (Fig. 1), and 4) almost two-thirds of adopters considered CCC cultivation as voluntary. The conclusion is that we consider most of the observed adoption as voluntary and due to agronomic reasons.

That said, 27% of adopters would grow less or no CCC without the EFA requirement (Table 5). Therefore, even if greening is not the primary reason for adoption for most adopters, it seems to play a role at least for some. The adoption intensities observed range up to 58%, but the median is only 12.5%, with 81% of farmers having adoption intensities of 26% or less. The EFA weighting factor for nitrogen-fixing crops is 0.7, which means that a farm would have to grow these crops on approximately 7% of its arable area. Thus, the EFA requirement may help explain why adoption intensities are often quite low.

Apart from this policy-related reason for limited adoption intensity, non-policy reasons are also relevant. Thirty- one percent of adopters affirmed that there were problems or risks preventing them from growing CCC on a larger area. The limiting factors cited by farmers include limited water availability and lack of profitability, although the number of adopters specifying the limiting factor(s) was rather small.

The large group of non-adopters frequently identified as the most important reason for non-adoption a lack of benefits from CCC, limited awareness (40% not aware of CCC) and knowledge, and lack of water. Twenty percent of non-adopters tried CCC in the past, but stopped mostly because it was not profitable and lots of work.

A lack of benefits as a barrier to wider adoption is also reflected by the fact that 68% of non-adopters indicate they would grow CCC if they were given subsidies for it. This figure is somewhat lower than in France and Romania (due to a higher prevalence of non-monetary constraints such as knowledge and water), but still substantial. The amount of subsidies required (WTA) has a mean of €169/ha (p25 = €70, p75 = €200). This is lower than the average direct payments from the CAP in Spain (around €250/ha, EC, 2018), and similar to the cost of cultivation reported by adopters (€177/ha, see Smit et al., 2019, p.29). There is no statistically significant difference in mean WTA from France or Romania.

### 3.2 Predicting non-adopters‘ responsiveness to subsidies

In the previous section we showed that high costs and lack of benefits are the most frequent reasons for non-adopters to not adopt CCC. This finding suggests that additional subsidies for CCC cultivation could turn non-adopters into adopters, which was confirmed by most non-adopters (74% in France, 80% in Romania, 68% in Spain). In this section we examine whether there are any farm or farmer characteristics that predict such responsiveness. The results may provide insights for designing better targeted interventions to increase CCC adoption (especially if these characteristics are easily observable). For example, if only larger farmers were responsive to subsidies, they could be targeted more effectively. Similarly, if WTA (amount of subsidies required to induce adoption) could be predicted by certain characteristics, the efficiency of public spending could also be enhanced. For example, some farmers may face lower costs and higher benefits (including stronger environmental beliefs and preferences) than others, so it may be hypothesised that these farmers require a smaller amount of subsidies (smaller WTA) for switching to adoption than others (Wuepper, 2019).

We use mean comparisons and correlational analysis to examine the relationships of a comprehensive list of farm characteristics with the two responsiveness variables of interest: First, we compare the mean values of the characteristics between non-adopters who would grow CCC if they received additional subsidies for it, and non-adopters who would still not grow CCC if additional subsidies were available (Table 6). Second, we examine the relationships of farm characteristics with the WTA among the subset of non-adopters responsive to subsidies (Table 7). Since WTA is a continuous variable, the mean WTA is compared across binary farm characteristics (e.g. mean WTA for a male vs. mean WTA for a female farmer) or correlations in the case of continuous farm characteristics (e.g. age). The analyses are done separately by country, which implies excluding the Netherlands because of the low number of non-adopters. All mean comparisons and correlations^6^ are tested for statistical significance. The list of farm characteristics includes first a set of classical farm and farmer characteristics such as farm size, types of farming operations, demographics and regions, among others. A second set comprises the importance farmers were asked to assign to potential reasons for non-adoption (with dummy variables taking on a value of 1 if the reason was assigned high or very high importance). The third set of farm characteristics are beliefs about the impact of CCC adoption on farm and environmental outcomes, as well as the farm impact of and farm influence on climate change. A full description of all variables is found in Table A2.

**Table 6:** Non-adopters‘ responsiveness to subsidies.

	Responsiveness to subsidies					
	Centre (France)		Sud-Muntenia (Romania)		Castile and Leon (Spain)	
	yes	no	yes	no	yes	no
	Mean (standard deviation)					
Farm size (ha)	127.71 (60.88)	113.09 (47.76)	71.97 (147.56)	81.27 (184.25)	110.56 (114.32)	92.05 (118.94)
Target crop size (ha)	100.55 (53.38)	83.55 (56.08)	65.79 (138.45)	77.6 (177.26)	97.78 (107.87)	74.35 (94.00)
CCC awareness (share of farmers)	0.87 (0.34)	0.91 (0.30)	0.42 * (0.50)	0.67 (0.50)	0.65 * (0.48)	0.48 (0.51)
Disadopter	0.39 (0.50)	0.55 (0.52)	0 (0)	0.07 * (0.26)	0.21 (0.41)	0.18 (0.38)
CCC information	0.45 (0.51)	0.18 (0.40)	0.19 * (0.40)	0 (0)	0.11 (0.32)	0.18 (0.38)
Permanent crops	0.55 (0.51)	0.36 (0.50)	0.11 (0.32)	0.07 (0.26)	0.11 (0.32)	0.05 (0.22)
Livestock	0.32 (0.48)	0.32 (0.48)	0.48 (0.50)	0.33 (0.49)	0.10 (0.30)	0.15 (0.36)
Grassland	0.26 (0.44)	0.45 (0.52)	0.02 (0.13)	0.07 (0.26)	0.10 (0.30)	0.15 (0.36)
Forests	0.03 (0.18)	0 (0)	0 (0)	0 (0)	0.06 (0.23)	0.10 (0.30)
Income (bracket)	1.87 (1.59)	1.27 (0.65)	1.89 (1.69)	2.40 (2.87)	2.10 (1.62)	1.93 (1.72)
Risk	5.42 (2.47)	4.55 (2.54)	6.45 (2.84)	7.00 (2.42)	4.70 (3.01)	4.15 (2.82)
Male	0.84 (0.37)	1 (0)	0.82 * (0.39)	0.6 (0.13)	0.97 (0.18)	1 (0)
Age	48.87 (11.29)	52.91 (6.56)	47.27 (12.78)	46.13 (17.13)	50.24 (11.26)	53.33 (11.22)
Education	3.16 (0.58)	2.91 (0.54)	3.02 (0.59)	3.2 (0.68)	2.10 *** (1.11)	1.58 (0.87)
Agricultural education	0.87 (0.34)	0.82 (0.40)	0.65 (0.48)	0.53 (0.52)	0.58 (0.50)	0.44 (0.50)
Private company	0.39 (0.50)	0.27 (0.47)	0.08 (0.27)	0.13 (0.35)	0.09 *** (0.29)	0.35 (0.48)
Other status	0.13 (0.34)	0.27 (0.47)	0 (0)	0 (0)	0.01 (0.11)	0 (0)
*Important reasons against adoption*						
Not rotation suitable	0.65 (0.49)	0.45 (0.52)	0.61 (0.49)	0.53 (0.52)	0.27 (0.45)	0.28 (0.45)
Pest/weed problem	0.35 (0.49)	0.09 (0.30)	0.19 (0.40)	0.13 (0.35)	0.16 (0.37)	0.20 (0.41)
No water available	0.29 (0.46)	0.45 (0.52)	0.55 (0.50)	0.60 (0.51)	0.38 (0.49)	0.31 (0.47)
Too costly	0.52 (0.51)	0.54 (0.52)	0.74 (0.44)	0.73 (0.46)	0.31 (0.47)	0.23 (0.42)
No labour available	0.16 (0.37)	0.27 (0.47)	0.44 ** (0.50)	0.73 (0.46)	0.06 (0.23)	0.13 (0.33)
No machinery available	0.03 (0.18)	0 (0)	0.55 (0.50)	0.67 (0.49)	0.09 (0.29)	0.05 (0.22)
No benefits	0.55 (0.51)	0.55 (0.52)	0.50 (0.50)	0.53 (0.52)	0.42 (0.50)	0.28 (0.45)
No awareness	0.13 (0.34)	0.09 (0.30)	0.40 (0.49)	0.53 (0.52)	0.33 (0.47)	0.38 (0.49)
Not confident	0.19 (0.40)	0.09 (0.30)	0.37 (0.49)	0.33 (0.49)	0.28 (0.45)	0.35 (0.48)
No seeds available	0.03 (0.18)	0.09 (0.30)	0.24 (0.43)	0.13 (0.35)	0.06 * (0.23)	0.15 (0.36)
Nobody does it	0.06 (0.25)	0 (0)	0.11 (0.32)	0.27 (0.46)	0.08 (0.27)	0.18 (0.38)
*Beliefs about farm-level impacts of CCC*						
Monetary benefit	0.26 (0.44)	0.18 (0.40)	0.03 (0.18)	0.07 (0.26)	0.21 (0.41)	0.18 (0.38)
Nonmonetary advantage	0.39 (0.50)	0.27 (0.47)	0.76 * (0.43)	0.53 (0.52)	0.48 * (0.50)	0.30 (0.46)
Reduce fertiliser use	0.32 (0.48)	0.36 (0.50)	0.24 (0.43)	0.33 (0.49)	0.62 (0.49)	0.55 (0.50)
Increase yield	0.06 (0.25)	0.18 (0.40)	0.23 (0.42)	0.27 (0.46)	0.61 * (0.49)	0.43 (0.50)
Increase soil carbon	0.71 * * (0.46)	0.36 (0.50)	0.23 (0.54)	0.33 (0.49)	0.90 *** (0.30)	0.70 (0.46)
*Beliefs about external environmental impacts*						
CCC benefit environment	0.58 (0.50)	0.36 (0.50)	0.65 ** (0.48)	0.33 (0.48)	0.79 (0.41)	0.65 (0.48)
CCC benefit climate change mitigation	0.26 (0.44)	0.18 (0.40)	0.48 ** (0.50)	0.20 (0.41)	0.52 (0.50)	0.38 (0.49)
Climate change harms farm business	0.68 (0.48)	0.45 (0.52)	0.82 ** (0.39)	0.53 (0.52)	0.55 ** (0.50)	0.33 (0.47)
Farm can make a difference to climate	0.29 (0.46)	0.09 (0.30)	0.56 *** (0.50)	0.13 (0.35)	0.49 (0.50)	0.45 (0.50)
No. of observations	31	11	62	15	89	40

*,**,*** significantly different from non-responsive farmers at the 10%, 5% and 1% level, respectively.

**Table 7:** Non-adopters‘ required amount of subsidies (WTA).

		How much subsidies (€)		
		Centre (France)	Sud-Muntenia (Romania)	Castile and Leon (Spain)
		Correlation or Mean (standard deviation)		
Farm size (ha)		-0.02	0.25 *	-0.14
Target crop size (ha)		0.12	0.25 **	-0.16
CCC awareness	yes	171.30 (101.37)	162.59 (13.28)	183.10 (178.59)
	no	205 (139.16)	148.46 (46.70)	143.39 (120.70)
Disadopter	yes	149.58 (95.74)	–	196.58 (158.24)
	no	192.11 (109.32)	154.38 (56.39)	161.86 (162.32)
CCC information	yes	176.07 (92.49)	180.42 (66.32)	275 ** (303.25)
	no	175.29 (116.73)	148.13 (52.58)	155.89 (130.73)
Permanent crops	yes	184.71 (117.05)	180.6 (95.35)	149 (93.98)
	no	164.71 (90.69)	151.05 (49.81)	171.84 (168.04)
Livestock	yes	161.5 (135.28)	169.89 ** (53.43)	225 (297.91)
	no	182.38 (89.88)	139.85 (55.99)	163 (139.87)
Grassland	yes	169.38 (138.06)	210 (-)	83.33 * (75.29)
	no	177.83 (94.24)	153.47 (56.39)	178.94 (165.64)
Forests	yes	100 (-)	–	290 * (400.62)
	no	178.17 (105.59)	154.38 (56.39)	162.08 (137.44)
Income (bracket)		0.04	0.14	0.02
Risk (1–10)		0.36 **	0.15	-0.17
Male	yes	181.73 (107.47)	152.27 (57.68)	169.36 (163.78)
	no	144 (92.90)	164.18 (51.28)	166.67 (57.74)
Age		-0.34 *	-0.11	-0.20 *
Education (1–4)		0.31 *	0.15	0.02
Agricultural education	yes	189.81 ** (102.77)	161.54 (57.98)	171.41 (182.84)
	no	80 (64.81)	141.37 (52.13)	167.6 (144)
Private company	yes	215.83 * (91.99)	160.02 (90.13)	181.25 (192.83)
	no	150.26 (106.59)	153.89 (53.67)	168.09 (159.11)
Other status	yes	152.5 (87.70)	154.38 (56.39)	400 (-)
	no	179.07 (108.8)	–	166.65 (160.20)
*Important reasons against adoption*				
Not rotation suitable	yes	189.75 (90.97)	145.45 (53.42)	254.17 *** (237.21)
	no	150 (126.81)	168.52 (59.18)	137.92 (108.66)
Pest/weed problem	yes	215.45 (113.96)	132.3 (57.29)	268.57 (270.38)
	no	153.75 (95.24)	159.68 (55.44)	150.73 (125.88)
No water available	yes	148.33 (95.98)	158.18 (51.10)	206.43 (213.32)
	no	186.82 (108.25)	149.77 (62.87)	152.21 (129.21)
Too costly	yes	206.25 * (92.94)	152.80 (52.60)	189.82 (148.20)
	no	143 (109.77)	158.94 (67.82)	159.84 (167.16)
No labour available	yes	188 (65.73)	150.5 (53.46)	232.00 (236.37)
	no	173.27 (111.54)	157.38 (59.14)	165.54 (156.87)
No machinery available	yes	100 (-)	160.28 (51.52)	268.13 * (199.96)
	no	178.17 (105.59)	147.22 (61.98)	159.51 (154.96)
No benefits	yes	216.47 ** (85.87)	145.37 (47.68)	223.78 *** (206.47)
	no	126.07 (106.88)	163.39 (63.43)	130.48 (105.15)
No awareness	yes	242.5 (109.05)	145.32 (51.86)	130.52 (119.70)
	no	165.74 (102.48)	160.51 (59.16)	188 (175.68)
Not confident	yes	236.67 (98.12)	162.07 (37.84)	156.8 (216.69)
	no	161 (102.75)	149.85 (64.95)	174.14 (135.37)
No seeds available	yes	100 (-)	159.6 (56.15)	214 (222.44)
	no	178.17 (105.59)	152.72 (56.97)	166.61 (158.24)
Nobody does it	yes	75 (35.36)	145.5 (27.40)	385.71 (313.20)
	no	182.59 (104.60)	155.51 (59.13)	150.79 (128.72)
*Beliefs about farm-level impacts of CCC*				
Monetary benefit	yes	105 (98.71)	126 (59.40)	164.57 (141.17)
	no	200.22 (97.05)	155.33 (56.56)	186.58 (224.15)
Nonmonetary advantage	yes	148.33 (110.69)	157.99 (47.55)	174.30 (184.58)
	no	192.89 (99.96)	143.08 (79.01)	164.57 (137.74)
Reduce fertiliser use	yes	141 (129.05)	136.08 (64.86)	189 (177.92)
	no	192.14 (89.90)	160.23 (52.84)	137.35 (125.68)
Increase yield	yes	35 ** (35.36)^1^	152.55 (71.78)	164.07 (170.91)
	no	185.34 (101.01)	154.92 (51.96)	177.29 (147.03)
Increase soil carbon	yes	163.18 (99.54)	139.65 (57.15)	174.12 (166.45)
	no	206.11 (116.93)	158.68 (56.03)	126.11 (99.62)
*Beliefs about external environmental impacts*				
CCC benefit environment	yes	146.39 * (111.72)	152.30 (50.60)	157.43 (157.76)
	no	216.15 (81.81)	158.17 (66.76)	212.89 (170.53)
CCC benefit climate change mitigation	yes	155 (126.72)	140.07 * (52.56)	185.87 (183.83)
	no	182.83 (98.22)	167.80 (57.32)	151.51 (132.72)
Climate change harms farm business	yes	174.76 (105.24)	159.6 (50.65)	147.14 (120.50)
	no	177.5 (109.37)	130.20 (76.06)	196.38 (198.48)
Farm can make a difference to climate	yes	131.11 (116.77)	145.26 (50.00)	147.27 (113.75)
	no	193.86 (96.36)	166.21 (62.71)	190.78 (195.88)
No. of observations (how much subsidies)		31	62	89

* ,**,*** correlation significant or difference between groups significant at the 10%, 5% and 1% level, respectively. ^1^ Note that only 2 farmers are in this category of non-adopters who believe that CCC increase yield.

Several variables show a statistically significant relationship with the likelihood that a non-adopter is willing to adopt CCC when offered additional subsidies (Table 6): In Spain, farmers responsive to subsidies tend to be more educated. Also in Spain, farmers responsive to subsidies are much less likely to have the judicial status of a private company than non-responsive farmers. In Romania, responsive farmers are much less likely to face a lack of labour availability. Some beliefs about farm-level impacts of CCC predict a higher responsiveness to subsidies. For example, responsive farmers are more likely to believe that CCC increase soil carbon in France and Spain. Some beliefs about external environmental impacts of CCC also appear to be related to responsiveness: In Romania, responsive farmers are more likely to believe that CCC benefit the environment and climate change mitigation, that climate change harms their farm business and that farmers can make a difference to climate, than unresponsive farmers. These differences are partly visible also in France and Spain, but mostly not statistically significant. Interestingly, several other variables are not correlated with reponsiveness to subsidies. Larger and higher-income farmers do not appear more or less likely to be responsive than smaller or lower-income farmers. Nor do risk preferences predict responsiveness.

Several statistically significant relationships were found regarding the amount of subsidies required for adoption (Table 7). Farm size is positively correlated with WTA in Romania. In Spain, farmers who receive information about CCC management have a higher WTA than farmers who do not. In France, WTA increases with willingness to take risk. Younger farmers in France and Spain tend to have a higher WTA. Romanian farmers for whom a lack of suitability of CCC for their crop rotation is an important reason for non-adoption require a larger amount of subsidies than farmers for whom it is not an important reason. Also, farmers in France and Spain who claimed that a lack of benefits from CCC was an important reason against adoption have a higher WTA. French farmers who believe that CCC would benefit the environment have a lower WTA, and Romanian farmers who believe that CCC benefit climate change mitigation also have a lower WTA. Generally, beliefs about external environmental impacts of CCC often predict sizeable differences in WTA, although these are mostly not statistically significant. A few other non-significant variables are worth highlighting: Again, income is not predictive of WTA. Also, beliefs about farm-level impacts of CCC are mostly insignificant.

In summary, these results suggest that there are few easily observable differences between responsive and unresponsive farmers, and that WTA is difficult to predict. This low predictability holds within countries, but also between countries, as relations between farm characteristics and responsiveness differ across countries.

## 4 Discussion

Environmental protection and climate change are becoming more prominent features of the discussion and design of policies affecting EU agriculture. The question of how policy can promote more environmentally and climate-friendly farming practices is central. Catch and cover crops (CCC) can have several beneficial effects on the environment, including soil fertility, soil health, erosion prevention, reduction of nutrient leaching, enhanced biodiversity and climate change mitigation. However, little is known about farmer adoption behaviour regarding CCC and its relation to policies. Our study contributes to this discussion with three main findings regarding the adoption rate, reasons for and against adoption, and the responsiveness of non-adopters to subsidies.

The first important finding concerns adoption rates and intensities. The estimated adoption rates were very high in Centre (84%) and Overijssel (99%), moderate in Sud-Muntenia (46%) and low in Castile and Leon (12%). The adoption rates in Centre and Sud-Muntenia were higher than expected, which is probably because the available Eurostat data was already several years old and some evolution has taken place. The farm adoption intensities were low and only ranged from 14% (Sud-Muntenia) to 20% (Overijssel).

Second, our examination of the reasons for adoption and adoption intensities involved multiple and sometimes contradictory observations, leading to results that were not always clear-cut. Nonetheless, the analysis revealed two principal groups of reasons: (obligatory) policies and (voluntary) agronomic motives. Environmental considerations were not found to be important drivers of adoption. Policy and agronomy have varying explanatory power in the different regions. There are two major policies that play a role: 1) the Nitrates Directive in the Netherlands and France, and 2) CAP greening (through the EFA requirement) in Romania and (to a lesser degree) France. Non-policy reasons have little relevance in France, the Netherlands and Romania. In Spain, policy is less important and agronomic reasons appear more important.

Differences in observed adoption rates (high in France and the Netherlands, moderate in Romania, low in Spain) can be mapped onto the underlying incentives: The Nitrates Directive, relevant in France and the Netherlands, makes CCC for many farmers obligatory, representing a driver of high adoption rates. Greening, relevant in France and Romania, is obligatory but CCC is only one (and not always the preferred) of the available EFA options. Hence, greening is only a moderately strong driver of adoption rates. Furthermore, farmers obliged to grow CCC under the Nitrates Directive often fulfill the EFA requirement without additional effort (deadweight). Both the Nitrates Directive and greening also help explain the low adoption intensities observed. Finally, non-policy reasons (agronomic in this case) only give a weak impetus for adoption, which can be observed in the low adoption rate in Spain.

Non-adopters in Centre, Sud-Muntenia and Castile and Leon provided a number of reasons for non-adoption, most frequently a lack of benefits and high costs, limited awareness/knowledge (especially in Spain), and unsuitability for crop rotations. Significant majorities of non-adopters in the three regions would be willing to start growing CCC if offered subsidies mostly in the range of €100–250/ha. The power of cost/profitability in shaping adoption emerging from our results is mirrored in the low adoption rates coinciding with cost concerns and lack of profitability reported for the US (Bergtold et al., 2019).

Our third finding concerns the responsiveness to subsidies among non-adopters. Given that we found some variability in responsiveness to subsidies, and especially in WTA, among non-adopters, we asked whether this variablity can be predicted (proxied) by any farm or farmer characteristics, which would enable more precise policy targeting. However, only few actionable proxies have been identified. For example, farm size does not seem to be related to responsiveness, and while it does predict higher WTA in Romania, it does not in the other countries. Younger farmers tend to have a higher WTA. It should be investigated further if some subgroups of farmers represent efficient targets for CCC-enhancing policies.

Beliefs about external environmental impacts, while not being easily observable farm characteristics, seem to be related to responsiveness and WTA (despite a few differences between countries and a lack of statistical significance in some cases). This finding is consistent with the idea that at least some farmers have a preference for environmental protection and climate change mitigation and, as a consequence, are willing to accept less money in exchange for the implementation of practices that may contribute to these ends. However, we have only observed correlations and a causal mechanism running in the opposite direction is also consistent with the data: that farmers for whom the cultivation of CCC is relatively unconstrained and cheaper are more likely to adopt beliefs that growing CCC has important positive externalities.

It should be noted that the results about responsiveness are based on stated preferences and hence may suffer from hypothetical bias and perhaps also strategic bias (where rent-seeking farmers report an inflated WTA). On the other hand, our survey data on the cost of cultivation for adopters with a mean of €144/ha (Smit el al, 2019, p.29) is roughly in line with the mean WTA of €165/ha (with France as an exception). In any case, our focus here was on predicting differences in responsiveness and WTA between farmers (which are less likely to be affected by such biases), rather than absolute levels.

## 5 Conclusions

The picture that emerges from this study is that CCC are not a practice inherently beneficial to farmers. Adopters mostly grow CCC because of policy obligations and incentives, and non-adopters are mostly open to adoption if policy provided stronger incentives. The data also suggests that environmental motives are of little relevance for explaining the observed CCC adoption patterns.

There are four main implications for policy from our results: First, there is room for higher adoption of CCC, in some regions more than in others. Second, most non-adopters would be willing to adopt, provided there are sufficiently large monetary incentives (or obligations with high penalties for non-compliance). Future research should examine whether the public benefits from CCC adoption are greater than the public costs of the required policy incentives. Third, given the lack of predictability of non-adopters‘ highly varied WTA with easily observed farm characteristics, it may be useful to consider WTA-revealing policy designs such as auctions (Latacz-Lohmann and Van der Hamsvoort, 1998). Finally, EU policymakers should take more seriously the possibility that different policies affecting farmers overlap and create deadweight, lowering the efficiency of public spending (Chabé-Ferret and Subervie, 2013).

## Figures and Tables

**Table A1:** Main reason for CCC adoption (% of adopters, by country).

	Centre (France)	Overijssel (Netherlands)	Sud-Muntenia (Romania)	Castile and Leon (Spain)
Obligation	60.0	52.3	66.7	11.5
Soil improvement (incl. organic matter)	26.7	46.3	–	26.9
N fixation	5.8	4.0	9.0	26.9
Subsidies	–	1.3	21.8	7.7
Improve next crop	4.2	2.7	–	19.2
Leaching reduction	6.7	12.8	–	–
Livestock feed	10.0	4.7	–	7.7
Weed management	1.7	2.0	–	7.7
Environmental reasons (incl. N)	1.7	0.7	–	7.7
More income	0.8	–	–	3.8
Soil cover	3.3	–	–	–
Improve N use	2.5	2.7	–	–
Benefits (unspecified)	2.5	–	2.6	–
Unclear reason	2.5	–	–	–
Aesthetic reasons	–	1.7	–	–
Biodiversity	1.7	–	–	–
Erosion reduction	1.7	–	–	–
Pesticide use reduction	1.7	–	–	–
Organic farming reason	1.7	–	–	–
Disease and pest management	1.7	–	–	–
Biogas	0.8	–	–	–
Undersow next crop	0.8	–	–	–
Nonconformism	0.8	–	–	–
No-till support	0.8	–	–	–
Hunting reasons	0.8	–	–	–
Follow maize	–	0.7	–	–
Catch water	–	0.7	–	–
n	120	149	78	26

Note: Main reason as spontaneously stated by farmer (open question, answers coded into dummies). Columns may add to more than 100% because farmers sometimes mentioned several reasons.

**Table A2:** Variables used for predicting responsiveness to subsidies.

Variable	Survey question	Unit
Responsiveness to subsidies		
Amount of subsidies required	How much subsidies would be necessary for you to start growing CCC?	€/ha
Farm size	How many hectares do you farm?	hectares
Target crop size	Out of these total farmed hectares, how many do you grow with cereals (wheat, barley, grain maize, triticale, rye, oats, spelt), oilseed rape, sunflower, soybeans or green maize/silage maize on average each year?	hectares
CCC awareness	Do you know what cover crops or catch crops are?	dummy
Disadopter	Have you ever grown CCC?	dummy
CCC information	Do you receive any information on CCC management?	dummy
Permanent crops	Which of the following activities do you have on your farm?^a^	dummy
Livestock		
Grassland		
Forests		
Income	In which income bracket is your household, including all income from all sources (farming and nonfarming) of all household members?	Ordinal from 0 to 200k in 25k increments, more than 200k
Risk	Are you generally a person who is willing to take risks or do you try to avoid taking risks?	Ordinal from 1 (not at all willing to take risks) to 10 (very willing to take risks)
Male	What is your gender?	1 = male, 0 = female
Age	What is your age?	years
Education	What is your education level?	Ordinal from 1 = primary school to 4 = university
Agricultural education	Do you have a special agricultural education?	dummy
Private company	Which is the farm's juridical status?^b^	dummy
Other status		
France	-*Baseline is the Netherlands*	dummy
Spain		
Romania		
Not rotation suitable	We would like to better understand why you do not grow CCC. Could you rate the importance of the following possible reasons for not growing CCC, and also rate the importance of any other reasons you may have in mind? From 1 (not important reason at all) to 5 (extremely important reason).	dummy (1 if reason was rated 4 or 5 in importance)
Pest/weed problem		
No water available		
Too costly		
No labour available		
No machinery available		
No benefits		
No awareness		
Not confident		
No seeds available		
Nobody does it		
Monetary benefit	Imagine you were growing CCC. Do you think you would make a monetary benefit (i.e. make money) or loss (i.e. lose money) from growing CCC?	dummy
Nonmonetary advantage	Consider that apart from a direct monetary benefit or loss, growing CCC can have advantages and disadvantages for you that are not monetary. Do you think that overall, the non-monetary advantages are greater or smaller than the disadvantages?	
Reduce fertiliser use	If you grew CCC, do you think you would need more or less fertiliser for the main crop grown after the CCC?	
Increase yield	If you grew CCC, do you think you would realise a higher or lower yield of the main crop grown after the CCC?	
Increase soil carbon	If you grew CCC, do you think it would increase or reduce soil organic matter / soil carbon, or not?	
CCC benefit environment	All things considered, do you think growing CCC on your farm would benefit or harm the environment, or not affect if significantly?	
CCC benefit climate change mitigation	All things considered, do you think growing CCC on your farm would benefit or harm efforts to mitigate (stop or slow) climate change, or not affect them significantly?	
Climate change harms farm business	Do you feel your farm business is benefiting or losing from climate change, or not being much affected by it?	
Farm can make a difference to climate	Do you think your farm in particular could make a significant difference to climate change mitigation; i.e. have an impact on how much climate change is occurring?	

a all farms have arable crops

b the baseline is 99% individual/family farms and 1% cooperatives.

## Annex A

See Table A1, Table A2.

## Footnotes

2 The distinction between constrained but beneficial adoption versus unconstrained but non-beneficial adoption is not universally applied in the literature. Sometimes the lack of profitability of non-beneficial adoption is referred to as a constraint/barrier to adoption (e.g. Sánchez et al., 2016; Roesch-McNally et al., 2018). An alternative distinction is between inability versus unwillingness to adopt (Snapp et al., 2005).

3 The minimum share is 5%, but different EFA options are subject to different weighting factors. The weighting factor for catch crops/green cover is 0.3, which translates into a minimum share of 5%/0.3 = 16.7% if that option is used. The weighting factor for nitrogen-fixing crops is 0.7, which translates into a minimum share of 5%/0.7 = 7.1%.

4 Albedo effects were also not taken into account, mainly because their mitigation potential is still uncertain and estimated to be relatively small relative to the contribution of carbon sequestration (Kaye and Quemada, 2017).

5 To enhance readability, we often use country names instead of region names. It should be kept in mind that the results are representative for the regions, not the whole countries

6 Note that we do not use regression methods or partial correlations because holding observed co-variates constant is not pertinent to our analysis: We do not seek to isolate statistical relationships of two variables from those with third variables (also, we do not seek to identify causal relationships). Instead, we examine whether individual, observable farm characteristics can predict (i.e. act as markers or proxies for) the not directly observable responsiveness to subsidies and WTA.
